# Post-response βγ power predicts the degree of choice-based learning in internally guided decision-making

**DOI:** 10.1038/srep32477

**Published:** 2016-08-31

**Authors:** Takashi Nakao, Noriaki Kanayama, Kentaro Katahira, Misaki Odani, Yosuke Ito, Yuki Hirata, Reika Nasuno, Hanako Ozaki, Ryosuke Hiramoto, Makoto Miyatani, Georg Northoff

**Affiliations:** 1 Department of Psychology, Graduate School of Education, Hiroshima University, Hiroshima, Japan; 2 Department of Psychiatry and Neurosciences, Institute of Biomedical & Health Sciences, Hiroshima University, Hiroshima, Japan; 3 Center of KANSEI Innovation, Hiroshima University, Hiroshima, Japan; 4 Department of Psychology, Graduate School of Environment, Nagoya University, Nagoya, Aichi, Japan; 5 Faculty of Education, Hiroshima University, Hiroshima, Japan; 6 Institute of Mental Health Research, University of Ottawa, Ottawa, Canada

## Abstract

Choosing an option increases a person’s preference for that option. This phenomenon, called choice-based learning (CBL), has been investigated separately in the contexts of internally guided decision-making (IDM, e.g., preference judgment), for which no objectively correct answer exists, and externally guided decision making (EDM, e.g., perceptual decision making), for which one objectively correct answer exists. For the present study, we compared decision making of these two types to examine differences of underlying neural processes of CBL. As IDM and EDM tasks, occupation preference judgment and salary judgment were used, respectively. To compare CBL for the two types of decision making, we developed a novel measurement of CBL: decision consistency. When CBL occurs, decision consistency is higher in the last-half trials than in first-half trials. Electroencephalography (EEG) data have demonstrated that the change of decision consistency is positively correlated with the fronto-central beta–gamma power after response in the first-half trials for IDM, but not for EDM. Those results demonstrate for the first time the difference of CBL between IDM and EDM. The fronto-central beta–gamma power is expected to reflect a key process of CBL, specifically for IDM.

How value is learned through the decision-making sequence is an important question that is explored broadly in psychology and neuroscience^1^,^2^,^3^,^4^,^5^,^6^. Many studies of decision making have used tasks in which a particular more-or-less predictable answer is available^7^,^8^,^9^,^10^. These are called externally guided decision-making tasks (EDM)^11^,^12^. In such situations, people must adjust their decision criteria to choose an externally defined single correct answer. Studies of decision making of this type have revealed that learning processes of item value have generally been explained by reinforcement learning theory, whereby the expected value (i.e., the magnitude of outcome times the probability of the outcome) guides the decision. The expected value is updated by the prediction error (i.e., discrepancies between expected and actual outcome)^13^,^14^,^15^. Recently, reports of studies using electroencephalography (EEG) have described that increased fronto-central beta–gamma power is visible after positive feedback in reward-based decision-making tasks. That increased power is thought to reflect positive reinforcement processes^16^,^17^,^18^,^19^,^20^,^21^,^22^,^23^,^24^.

In addition to EDM, other types of decision making in which no objective correct answer is available^25^,^26^,^27^,^28^,^29^,^30^,^31^. They constitute internally guided decision making (IDM)^11^. Such decisions are usually made in the context of preference judgment^32^,^33^,^34^,^35^ as well as in the context of moral judgment^36^,^37^,^38^, where the answer depends on the subject’s own, i.e., *internal*, preferences rather than on circumstantial, i.e., *external*, criteria. Although no prediction error is available in the IDM, the value or preference of an item is known to be altered through the results of a person’s own decision. The preference for the chosen item is increased, but that for the rejected item is decreased^39^,^40^,^41^,^42^,^43^. This phenomenon is designated in the literature as choice-induced preference change^39^,^41^,^44^,^45^.

Recently, as with the EDM, a reinforcement-learning-like mechanism is thought to explain how choice-induced preference change occurs^46^,^47^,^48^. Akaishi *et al.*^46^ reported that people tend to repeat the same decision when required to make a decision based on perceptually ambiguous stimulus with no feedback. Moreover, Akaishi *et al.*^46^ demonstrated that the underlying mechanism of that tendency is explained well by a reinforcement-learning-based model in which the likelihood of choosing an option is updated by the choice itself, called choice-based learning (CBL). Although the perceptual decision-making task used by Akaishi *et al.*^46^ was more of EDM than IDM, they proposed that the same mechanism can explain the choice-induced preference change: in a situation where no externally defined single correct answer exists, choosing a particular item is followed by reinforcement of the chosen item as if the own choice is regarded as a correct answer. This process engenders an increase in the preference for the chosen item and an increase in the likelihood of choosing the same item at the following opportunity. Consistent with the proposal by Akaishi *et al.*^46^, neuroimaging studies of choice-induced preference change have shown activation within typical neural substrates of reward-based reinforcement learning (e.g., ventral striatum, dorsal anterior cingulate cortex, and ventromedial prefrontal cortex)^40^,^49^,^50^,^51^,^52^. However, those earlier studies examined brain activities after stimulus onset for free choice between two items^50^,^52^ or for preference ratings of respective items^40^,^49^,^51^ using functional magnetic resonance imaging (fMRI). Consequently, although neural activities related to choice response are expected to play a crucially important role in CBL because the choice itself induces the value change, the relation between CBL and neural activities related to the response remains unclear.

Unfortunately, IDM and EDM have been investigated using different paradigms in terms of stimuli, tasks^11^, and means to measure the change of preference or value through decision making. In IDM studies, choice-induced preference change has been investigated using the free choice paradigm^43^, which includes pre-rating, free choice, and post-rating phases. In the pre-rating phase, the participant rates several items according to the participant’s own preferences. In the free choice phase, two items are presented. Participants are asked to choose the preferred one. In the post-rating phase, a participant is asked to rate all items again in the same way as with pre-ratings. In this paradigm, decision making (i.e., free choice) for a particular item is required once. Then the value change is measured by subjective rating^39^,^41^,^45^. In contrast, in EDM studies, decision making for the same stimuli is repeated several times to ascertain the choice-induced change in the likelihood of choosing the stimuli at the subsequent opportunity^7^,^8^,^46^,^53^. Those differences have made it difficult to compare decision making of these two types. Consequently, no report in the relevant literature has described comparison of CBL between these two types of decision making within the same paradigm. For that reason, the differences between the decision making types in terms of the underlying neural processes of CBL remain to be elucidated.

This study was undertaken to compare IDM to EDM in terms of neural activities around choice response related to CBL. To compare CBL of these two types of decision making, we developed a novel measurement, i.e., the change of decision consistency, which is applicable to both IDM and EDM tasks (for more details, see Fig. 1(c) and *Methods*). The decision consistency is expected to be higher in the last half of the sequence of decision making than in the first half if the likelihood of choosing a chosen item is increased, or if the likelihood of choosing a rejected item is decreased. By conducting simulation using a simple CBL model, we confirmed that an increase of decision consistency was observed when CBL occurs (for details, see Supplemental Information and Fig. S1(c–e) for Simulation 2). Although the free choice paradigm is known to have a methodological flaw by which choice-induced preference change is observed even if no preference change exists^42^,^44^,^54^, the change of decision consistency can avoid that problem. We also confirmed this point by conducting a simulation (Supplemental Information and Fig. S1(a,b) for Simulation 1). Actually, we expect that increased decision consistency is observed both in IDM and in EDM because CBL has been observed in IDM (i.e., choice-induced preference change)^39^,^41^,^45^ and EDM^46^.

Additionally, we expect that a difference of CBL between IDM and EDM is observed in the relation between the change of decision consistency and the neural activities related to the response. Although no strong activation within the reward-related neural substrates was reported in CBL of EDM^46^, it was reported in CBL of IDM^40^,^49^,^50^,^51^,^52^. This evidence suggests that CBL in IDM is associated with reinforcement processes such as reward-based reinforcement learning, different from CBL in EDM, even if the item is not visibly reinforced by the externally delivered reward. For measurement of neural processes related to choice response, we specifically examined the beta–gamma (14–60 Hz) power for the response. As described above, earlier studies of reward-based decision making showed increased beta–gamma power to around 200–500 ms after presenting positive feedback. That range is thought to reflect positive reinforcement processes^16^,^17^,^18^,^19^,^20^,^21^,^22^,^23^,^24^. Participants who showed greater beta–gamma power around 200–500 ms after response in the first-half trials are expected to show more CBL effects (i.e., increased decision consistency in the last-half trials than in first-half trials) in IDM, but not in EDM.

## Results

### Validation of the occupation preference judgment task as IDM, and salary judgment task as EDM

**Figure 1:**
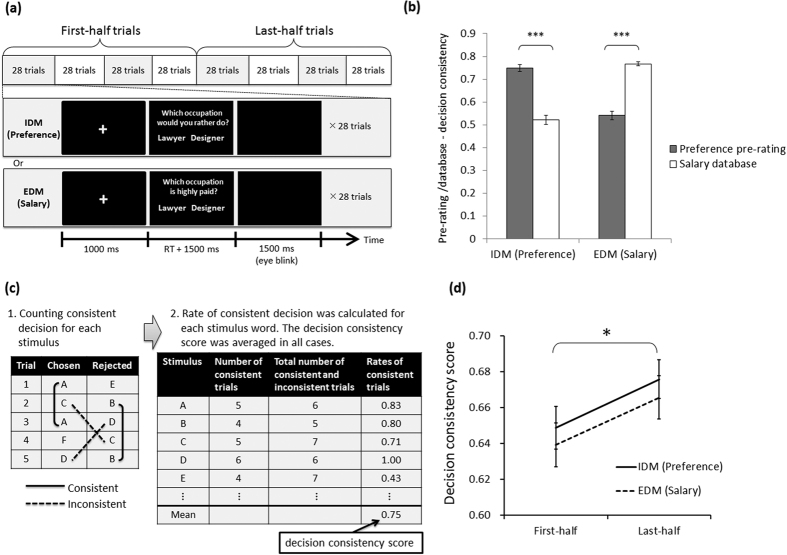
(**a**) Design of experimental tasks. As the internally guided decision making (IDM) and externally guided decision making (EDM) tasks, we used occupation preference judgment^12^,^34^, for which no objectively correct answer exists, and salary judgment for which one objectively correct answer exists, respectively. Participants performed counterbalanced tasks of two types. RT denotes the reaction time. (**b**) Pre-rating–decision consistency (i.e., how often participants’ decisions of preference judgment were consistent with pre-ratings of the occupational preference), and average annual salary database–decision consistency (i.e., how often participants’ decisions were consistent with the actual average annual salary, which is based on a statistical survey by the Ministry of Health, Labour and Welfare of Japan) for each decision-making task (see Supplemental Information for more detailed methods to calculate these indexes). ***Denotes a significant main effect of epoch (*p* < 0.0001). Error bars show standard errors. (**c**) Schematic figure of the calculation of decision consistency score. The decision consistency score represents the rate of trials in which a certain occupation word was chosen or rejected repeatedly. In cases where a participant chose A (first trial in the example of this figure) and it was chosen again in the trial in which A was presented the next time (third trial in this example), we counted that trial as a consistent decision. In addition, in cases where a participant rejected B (second trial in this example) and it was rejected again in the trial in which B was presented the next time (fifth trial in this example), we counted that trial as a consistent decision. The consistent decisions were counted for each occupation word. Then that number was converted to a rate of consistent decision by dividing the number of consistent decisions by the sum of the number of consistent and inconsistent decisions. The average of the rate of consistent decisions was calculated across all occupation words. (**d**) Decision consistency scores for the first-half and the last-half trials in IDM (preference judgment) and EDM (salary judgment) tasks. *Denotes a significant main effect of epoch (*p* < 0.01). Error bars show standard errors.

As IDM and EDM tasks, we respectively used occupation preference judgment^12^,^34^ (e.g., “Which occupation would you rather do? …Lawyer or Designer”), for which no objectively correct answer exists, and salary judgment (e.g., “Which occupation is highly paid? …Lawyer or Designer”) for which one objectively correct answer exists (Fig. 1(a)). To confirm the decision criteria for each task, we computed the pre-rating–decision consistency (i.e., how often participants’ decisions of preference judgment were consistent with pre-ratings of the subjective occupational preference; see Supplemental Information for detailed methods), and average annual salary database–decision consistency (i.e., how often participants’ decisions were consistent with the actual average annual salary, which is based on a statistical survey by the Ministry of Health, Labour and Welfare of Japan; see Supplemental Information for details). As Fig. 1(b) shows, results of these indexes confirmed that the occupation preference judgment was based on internal criteria (i.e., subjective occupation preference) different from the salary judgment, whereas the salary judgment was based on the external criteria (i.e., the average salary of the presented occupation) different from the preference judgement. Two-way repeated-measures ANOVA (two decision tasks (IDM, EDM) × two criteria (subjective occupation preference, the average salary of the presented occupation) revealed a significant interaction (*F*(1, 23) = 124.85; *p* < 0.001, *η*_*g*_^2^ = 0.64). Post-hoc tests of the interaction revealed that the pre-rating–decision consistency was higher in the IDM (preference judgment) task than in the EDM (salary judgment) tasks (*p* < 0.001) in the dimensions of subjective occupation preference. For the dimension of average annual salary, the average annual salary database–decision consistency was higher in the EDM (salary judgment) task than in the IDM (preference judgment) task (*p* < 0.001). These results indicate that participants used internal or external criteria, respectively, for the preference judgment and salary judgment.

### Decision consistency

The decision consistency score represents the ratio of trials in which a certain occupation word was repeatedly chosen or rejected (for details see Fig. 1(c) and *Methods*). Figure 1(d) presents a summary of the mean decision consistency score. It shows that decision consistency was higher in the respective last-half trials than in the first-half trials both in IDM (preference judgment) and EDM (salary judgment) tasks. Consistent with this observation, two-way repeated-measures ANOVA (two decision tasks (IDM, EDM) × two epochs (first-half and last-half trials)) revealed a significant main effect of epoch (*F*(1, 23) = 8.20, *p* = 0.009, *η*_*G*_^*2*^ = 0.05). No significant main effect of decision task (*F*(1, 23) = 0.50, *p* = 0.49, *η*_*G*_^*2*^ = 0.01) or interaction (*F*(1, 23) = 0.001, *p* = 0.97, *η*_*G*_^*2*^ = 0.00) was found.

Regarding the results of the trial-based decision consistency score, reaction times, and pre-rating data including subjective rating for average annual salary, see Supplemental Information (Supplementary Fig. S3).

### Relation between the change of decision consistency and post-response beta gamma power

**Figure 2:**
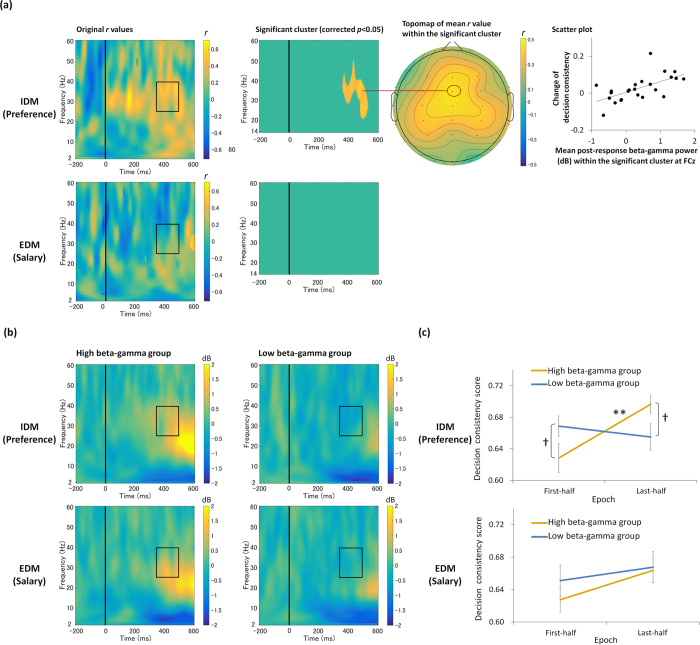
(**a**) Correlation results between the response-locked event-related spectral perturbations (ERSP) at FCz for the first-half trials and the change of decision consistency (last-half trials–first-half trials) for each decision-making task (see also Fig. S4 for similar results obtained using pre-stimulus baseline corrected response-locked ERSP data). Scalp topography of the mean *r*-value within the significant cluster for the preference task, and the scatter plot between the change of decision consistency and mean beta–gamma power within the significant cluster are shown on the right side. (**b**) ERSP images at FCz for the first-half trials of IDM (preference) and EDM (salary) tasks are shown separately for high and low beta–gamma groups of each task. These figures are presented for illustration purposes. (**c**) Decision consistency for the first-half and the last-half trials in IDM (preference) and EDM (salary judgment) tasks shown separately for high and low beta–gamma groups. ^†^Denotes a marginal difference of *p* < 0.10. **Denotes a significant difference of *p* < 0.005. Error bars represent standard errors. Rectangles presented in the original *r*-values for preference task (**a**) and ERSP images (**b**) show the time-frequency window used for calculating the average power for dividing high and low beta–gamma groups. Internally guided and externally guided decision-making are denoted respectively as IDM and EDM.

Figure 2(a) presents correlation results between the event-related spectral perturbations (ERSP) in the first-half trials at FCz and the change of decision consistency in each task. Positive correlation was found in the beta–gamma (25–40 Hz) range after response in IDM (preference judgment), but not in EDM (salary judgment).

Permutation tests, which are used to avoid multiple comparisons in the large time–frequency space (see *Methods* and Supplemental Information for more details), yield significant positive correlation between the beta–gamma power around 400 ms after response in the IDM task (cluster *r*-value sum = 271.67, cluster count = 582, corrected *p* < 0.05). This result indicates that participants who showed greater beta–gamma power around 400 ms relative to the pre-response baseline during the first-half trials show more increased decision consistency in last-half trials than in first-half trials. No such significant correlation was found for the EDM task.

We conducted the following additional analysis to ascertain whether the increase of decision consistency in the IDM task is observed in participants who showed increased post-response beta–gamma power in the first-half trials, or not. We divided participants into two groups (12 participants for each group) based on the median of the averaged post-response beta–gamma power (350–500 ms and 25–40 Hz) at FCz (see Fig. 2(b)). Figure 2(c) presents the respective decision consistency scores of the IDM task for these two groups in the first-half and the last-half trials. Two-way mixed ANOVA (groups of beta–gamma power in the first-half trials (high beta–gamma group, low beta–gamma group) × two epochs (first-half and last-half trials)) revealed a significant main effect of epoch (*F*(1, 22) = 5.604, *p* = 0.03, *η*_*G*_^*2*^ = 0.07), and significant interaction (*F*(1, 22) = 12.75, *p* = 0.002, *η*_*G*_^*2*^ = 0.14). Shaffer post-hoc test results revealed that the decision consistency was higher in last-half trials than in the first-half trials in the high beta–gamma group (*p* = 0.004). No such difference was found in the low beta–gamma group. In the first-half trials, the decision consistency was marginally lower in the high beta–gamma group than the low beta–gamma group (*p* = 0.09). In the last-half trials, the decision consistency was marginally higher in the high beta–gamma group than the low beta–gamma group (*p* = 0.06). These results indicate that the increase of decision consistency in the IDM task was observed only in the participants who showed increased post-response beta–gamma power in the first-half trials. Although we conducted the same comparisons for the EDM task by dividing the high and the low beta–gamma groups (12 participants for each group), no significant difference was found (see Fig. 2(b,c)).

Regarding the results of averaged ERSP across all participants, see Supplemental Information (Supplementary Fig. S5).

The gamma power is known to show cross-frequency coupling with theta–alpha phase^55^,^56^. Therefore, we examined cross-frequency phase-amplitude coupling between the beta–gamma power and lower theta–alpha phase at FCz. Results obtained by application of permutation-corrected *p* < 0.05 show no significant coupling (see Supplemental Information and Fig. S6 for methods and detailed results).

## Discussion

This study was conducted to compare IDM and EDM within the same paradigm in terms of CBL and underlying neural processes. Regarding CBL measured as the increase of decision consistency, significantly higher decision consistencies in the last-half trials than in the first-half trials were found for both IDM (i.e., occupation preference judgment) and EDM (i.e., salary judgment) tasks (Fig. 1(d)), as we had expected. These results were consistent with those obtained in previous studies of CBL conducted using the perceptual-decision-making task^46^ and the free choice paradigm^39^,^41^,^45^.

Differences of CBL between IDM and EDM were observed in relation to the underlying neural activities (Fig. 2). In IDM, participants with increased fronto-central beta–gamma power after response (approximately 400 ms) in the first-half trials showed increased decision consistency. The post-response beta–gamma power predicted the subsequent change of decision consistency as the measurement of CBL. No such relation was found for EDM. These data are the first demonstrating a difference of CBL between IDM and EDM in terms of the beta–gamma activities after response. The post-response beta–gamma is thought to reflect a key process in CBL of IDM.

Then, what is the difference between the underlying mechanisms of IDM and EDM? One plausible difference is whether a reward-related process is recruited or not. Regarding IDM, correlation between the beta–gamma power and the change of decision consistency might reflect the reward-based learning process. As described above, the front–central beta–gamma power after positive feedback is thought to reflect positive reinforcement processes^16^,^17^,^18^,^19^,^20^,^21^,^22^,^23^,^24^. Although the beta–gamma power in the present data was observed after response instead of positive feedback, the time range and scalp distribution of the present beta-gamma activity show similar physiological properties to those of the reward-based decision making tasks^16^,^17^,^18^,^19^,^20^,^21^,^22^,^23^,^24^. This evidence implies that reward-based learning process takes part in the CBL of IDM. This interpretation seems reasonable given that the neuroimaging studies of CBL have reported activation within neural substrates related to reward-based reinforcement learning^40^,^49^,^50^,^51^,^52^. In contrast, regarding EDM, Akaishi *et al.*^46^ reported that CBL is probably mediated by a non-reward based learning process. Considering that the beta–gamma power is related to the positive reinforcement process^16^,^17^,^18^,^19^,^20^,^21^,^22^,^23^,^24^, the present results of EDM (i.e., no significant relation between the beta–gamma power and CBL) are consistent with results reported by Akaishi *et al.*^46^, who reported no strong activation within the reward-related neural substrates.

When we calculated the index of the CBL at the trial level (trial-based decision consistency; see Supplemental Information including Figs S2 and S3(a)), a change of decision consistency was observed in the IDM but not in EDM. This behavioral result also suggests that the underlying mechanisms of CBL differ between IDM and EDM. Different from the results of epoch-based decision consistency (Last-half–first half; Fig. 1(d)), the increased decision consistency was observed only in the IDM. A possible reason for the discrepancy is that trial-based decision consistency can be affected strongly by the combination of stimulus: if participants selected “Lawyer” in the trial of “Lawyer” vs. “Designer” for preference judgment, the choice would be affected if the more preferred or less preferred occupation were paired with “Lawyer”, whether the “Lawyer” is chosen or rejected at the next opportunity. The combination of the two options was determined randomly across trials. Therefore, if no CBL exists, then no change of decision consistency is observed (this result was confirmed by the Simulation 1 reported in Supplemental Information and Fig. S1(a,b)). However, that effect from the combination of the two options functions as noise to decrease the statistical power to observe decision consistency (both in the epoch-based and trial-based decision consistency). Moreover, trial-based decision consistency would be affected strongly by that noise because fewer trials were used to calculate one index value. It is noteworthy that, even with such trial-based decision consistency, increased decision consistency remained for IDM. This result reflects that the CBL in IDM, which might be induced by reward-based learning, is robust compared to that in EDM, which is thought be induced by non-reward based learning.

The increase of decision consistency in the IDM task was observed only for participants who showed increased post-response beta–gamma power in the first-half trials (Fig. 2(c)). This result suggests that performing IDM is insufficient for inducing CBL, and suggests that the increased beta–gamma power after response is necessary. Not only external factors (e.g., type of decision-making task, IDM or EDM) but also the internal factors of participants (e.g., individual difference in reactivity of reward-related process for the specific IDM task, which is reflected in the beta–gamma power) can affect the degree of CBL in IDM. Izuma and Murayama^42^ reported from their meta-analysis that the effect size of choice-induced preference change was small. Our results imply that a possible reason for the small effect size is the individual difference of the reactivity for the specific preference task and stimulus. Further studies must be conducted to examine internal factors reflected in the beta–gamma activity to elucidate CBL in IDM.

Despite the importance of our data for revealing the relations between the beta–gamma powers and CBL in IDM, these findings leave several questions unresolved. First, we specifically examined beta–gamma activities based on the notion that the beta–gamma reflects positive reinforcement processes in reward-based learning tasks^16^,^17^,^18^,^19^,^20^,^21^,^22^. Moreover, the reward-related neural substrates are known to be activated in relation to the choice-induced preference change^40^,^49^,^50^,^51^,^52^. However, because we did not manipulate and measure the amount of reward during the IDM (preference) task, we cannot conclude that the beta–gamma power observed in the present study reflects the reward-related activities as it did in the case of the reward-based reinforcement learning task. Additional studies must be conducted to clarify the functional role of beta–gamma power at the neuronal and psychological levels.

Second, we used the change of decision consistency as the measure of CBL because that index can avoid the methodological flaw of the free choice paradigm^42^,^44^,^54^ and because it is applicable for both IDM and EDM tasks. However, we cannot judge whether the increase of decision consistency results from increasing the value of chosen items or decreasing the value of rejected items. Choice-induced preference change is well known to be observed as an increased preference for chosen items and/or decreased preference for rejected items^39^,^40^,^41^,^42^. The increase of decision consistency is visible by one or both possibilities (see Fig. S1(c–e)). To specify the relation between the reinforcement process and beta–gamma power in the CBL of IDM further, it is necessary to conduct studies to measure the index of choice-induced preference change (i.e., change of subjective preference rating for each item) without suffering from the methodological flaw of the free-choice paradigm^42^,^44^,^54^.

Third, we used salary judgment as the EDM task by confirming that the participants’ decisions were consistent with the external criteria (i.e., actual average annual salary based on a statistical survey by the Ministry of Health, Labour and Welfare of Japan; see Fig. 1(b)). Nevertheless, the salary task differed from the EDM tasks used in earlier studies (i.e., reward based^8^,^9^,^10^,^13^ and perceptual decision making^46^). Therefore, one must be careful when generalizing the present result to any kind of EDM task.

Fourth, using simulations (Fig. S1(c–e)) we confirmed an increase of decision consistency when CBL occurs. However, we cannot exclude the possibility that other models can explain the increase of decision consistency better than the model used for the present simulation (Simulation 2, see Supplemental Information). For example, a lower decision noise level in the last-half trials than in the first-half trials can increase the decision consistency, even if no true preference change occurs. This possibility is apparent in our simulation presented in Fig. S1(a): higher decision consistency was observed where less decision noise was included. Although the decision noise level was constant in that simulation, the noise level can be decreased in the last-half trial. Reduction of the decision noise can be interpreted as reflecting that people become able to recognize their own preferences well via a series of decisions. This can be regarded as a kind of CBL. However, this point differs from the explanation assumed for previous CBL studies^39^,^40^,^41^,^46^,^51^,^52^. It remains unclear which of the true preference changes or reduction of decision noise is the better model for the CBL.

In summary, we first compared CBL in IDM and EDM within the same paradigm. Differences of CBL between these two types of decision making were observed in relation to the underlying neural activities. The beta–gamma power around 400 ms after response predicted the following change of decision consistency in IDM, not in EDM. Although the timing of reinforcement process in EDM (i.e., reward-based decision making) has been well identified, it has been less than clear in IDM. This study provides the first evidence that neural activities after free choice response are related to CBL in IDM. The post-response front-central beta–gamma power is thought to reflect a key process of learning based on one’s own decision in IDM.

## Methods

### Participants

This study examined 24 healthy undergraduate students (11 male; age range = 18–21 years, mean age = 19.58 years) recruited from Hiroshima University. All participants were native Japanese speakers, right-handed, with normal or corrected-to-normal vision. All were free of neurological and psychiatric disorders. No participant was either medicated or a habitual drinker or smoker. All experimental protocols were conducted in accordance with guidelines approved by the Ethical Committee of the Graduate School of Education, Hiroshima University. Written informed consent was obtained from each participant before the investigation. Each participant was paid a small fee for participation.

### Stimuli and Tasks

As in previous studies^12^,^31^, 28 occupation-related terms (e.g., lawyer, carpenter) were collected from the Classification of Occupation for Employment Security Service (ESCO: http://www.jil.go.jp/institute/seika/shokugyo/sakuin/). One term was chosen from each of the third categories of the ESCO. To determine the correct response of the EDM (salary judgment) task, the average annual salary for each occupation was collected from Nensyu-labo (http://nensyu-labo.com/2nd_syokugyou.htm), the salary data of which were based on a statistical survey by the Ministry of Health, Labour and Welfare of Japan. The average annual salary did not covariate with word-length and used-frequency of each occupation-related term (for details, see Supplemental Information). The term pairs for the IDM (occupation preference judgment) and the EDM (salary judgment) tasks were produced. For each participant and task, 112 pairs were generated randomly with the restriction that each term was used eight times.

In the IDM (occupation preference judgment) task, the two occupation words were presented. Then participants were asked to judge which occupation they would rather do (“Which occupation would you rather do?”) by pressing the button on the corresponding side, as in earlier studies^12^,^34^ (see Fig. 1(a)). Participants were instructed explicitly that no objectively correct answer exists: they must make their own decisions.

In the EDM (salary judgment) task, participants were asked to judge which is a highly paid occupation on average (“Which occupation is highly paid?”) by pressing the button on the corresponding side (see Fig. 1(a)). Participants were instructed clearly that the average salary is based on the statistical survey by Ministry of Health, Labour and Welfare, and that there was one objective correct answer. Following the method described by Akaishi *et al.*^46^, we did not present the visual feedback in the EDM task to examine the CBL.

Details of the procedures used for these two tasks are presented in the following sections describing procedures.

### Procedure

When participants arrived in the laboratory, the experimental procedure was explained. They read an information sheet and signed the consent form. After assigning ratings for each occupation term (for details, see Supplemental Information) and electrode placement, participants were seated on a comfortable chair facing a computer screen in a quiet electrically shielded room. A chin rest was used to help participants maintain the head position during recording.

Participants performed counterbalanced tasks of two types. Four blocks of 28 trials were conducted for each task (see Fig. 1(a)). The presentation side of words was randomized across participants. The order of trials was also randomized. Before the experimental trials, participants were given three practice trials for each task to familiarize them with the tasks.

Each block began with the appearance of an instruction related to the task type on the screen for 3,000 ms. After a 1,000 ms blank, trials began: the fixation cross was presented for 1,000 ms. Then two stimulus words and a question (“Which occupation would you rather do?” or “Which occupation is highly paid?”) were presented. The stimuli and question remained visible on the screen until 1,500 ms after the participant pressed the button. The stimuli and question were replaced with the blank screen, which was presented for 1,500 ms. While this blank screen was displayed, participants were allowed an eye blink. After this blank-screen period, the subsequent trial began (the fixation cross was presented for 1,000 ms). The reaction time (RT) from the presentation of the stimuli to the response was recorded. Participants were instructed to press either the left or right button with the corresponding index finger as quickly and accurately as possible after each stimulus was presented. Additionally, they were asked to avoid eye blinking during times other than the blank screen.

### Decision consistency score

To measure the degree of CBL, the decision consistency scores were calculated, respectively, for the first-half and the last-half trials (see Fig. 1(c)). This index can avoid the methodological flaw of the free choice paradigm (see also Fig. S1(a,b), Simulation 1)^42^,^44^,^54^. In addition, the index is applicable for both the IDM and EDM tasks. The decision consistency score represents the rate of trials in which a certain occupation word was repeatedly chosen or rejected. In cases where a participant chose an occupation in a certain trial and it was chosen again in the trial in which the occupation was presented the next time, we counted that trial as a consistent decision. In addition, in cases where a participant rejected an occupation in a certain trial and it was rejected again in the trial in which the occupation was presented the next time, we counted that trial as a consistent decision. The consistent decisions were counted for each occupation word. Then that number was converted to a rate of consistent decision by dividing the number of consistent decisions by the sum of the number of consistent and inconsistent decisions. The average of the rate of consistent decisions was calculated across all occupation words. That figure was used as the decision consistency score.

### EEG recordings and analyses

EEG was recorded using 30 silver–silver chloride cup electrodes attached to an electrocap (Quik-Cap; NeuroScan). The signals were amplified with a bandpass of 0.0159–120 Hz and were digitized at a 1,000 Hz sampling rate using the EEG recorder (EEG-1100; Nihon Kohden Corp., Tokyo, Japan). Supplemental Information provides more details of EEG recording.

EEG data analysis was conducted using EEGLAB toolbox^57^ running under Matlab 8.4.0 (The Mathworks Inc.). Data were filtered using a low-pass filter of 120 Hz and a high-pass filter of 1 Hz. Response-locked data epochs starting from 1,000 ms before and 1,500 ms after the response button press were extracted. Baselines for event-related potential (ERP) were taken from −500 ms to −250 ms relative to the response onset. Independent component analysis (ICA) was used for artifact rejection from EEG data (for details, see Supplemental Information). After these artifact rejection processes, each type of data epoch was divided into experimental conditions.

Event-related spectral perturbation (ERSP) is the degree to which the spectral power differs from the mean baseline power as a function of time and frequency. For ERSP calculation, the Morlet wavelet was used. Starting from −442 ms before and ending up to 943 ms following response onset, 59 linear-spaced frequencies ranging from 2 Hz to 60 Hz were calculated every 3 ms. The wavelet cycle was increasing from 2 cycles at 2 Hz to 8 cycles at 60 Hz. The pre-response duration (−442–0 ms) was used as the baseline as reported by Wang *et al.*^58^ because CBL is expected to be related to the change of neural activity from before to after decision making. We avoided using the pre-stimulus baseline for results reported in the main text because post-response activities (i.e., the target activities of the present study) of the preceding trial can contaminate the pre-stimulus baseline^59^. However, to confirm the correlation result observed in the post-response duration was not caused by the pre-response baseline activity, this report presents the correlation results obtained after applying the pre-stimulus baseline to the response-locked ERSP in Supplemental Information (Fig. S4).

Regarding statistical analyses of group averaged ERSP, see Supplemental Information.

### Correlation analyses

To ascertain whether the participants who showed greater beta–gamma power during the first-half trials show a stronger CBL effect or not (i.e., increased decision consistency in last-half trials than first-half trials), the difference of decision consistency (last half minus first half) was calculated for each participant and task. The response-locked ERSPs of first-half trials of each task at FCz were used for these analyses. Data falling outside mean ± 3 *SD* were regarded as outliers, and were removed. We performed cluster-based permutation tests^60^ to avoid issues related to multiple comparisons in the large time–frequency space. For the permutation test, the frequency range and time window were limited, respectively, to the beta–gamma band (14–60 Hz) and −200 to 600 ms (for details see Supplemental Information).

## Additional Information

**How to cite this article**: Nakao, T. *et al.* Post-response βγ power predicts the degree of choice-based learning in internally guided decision-making. *Sci. Rep.* **6**, 32477; doi: 10.1038/srep32477 (2016).

## Supplementary Material

Supplementary Information
