# Supplementary material for: Post-response βγ power predicts the degree of choice-based learning in internally guided decision-making
Source: Sci Rep. 2016 Aug 31;6:32477. doi: 10.1038/srep32477 (PMC5006019; doi:10.1038/srep32477)
Supplement: Supplementary Information [file srep32477-s1.pdf]

Supplemental information

Post-response βγ power predicts the degree of choice-based learning in internally guided decision-making

Takashi Nakao, Noriaki Kanayama, Kentaro Katahira, Misaki Odani, Yosuke Ito, Yuki Hirata, Reika Nasuno, Hanako Ozaki, Ryosuke Hiramoto, Makoto Miyatani, Georg Northoff

Simulation of decision consistency

Simulation 1

We conducted a simulation (Simulation 1) to confirm that the decision consistency is unaffected by the problem described by Chen and Risen [1], in which choice-induced preference changes are apparent in the free choice paradigm, even if no preference change exists. One cause of this problem is the use of noise-contaminated subjective ratings to assess the preference change. The change of decision consistency can avoid that problem by avoiding use of pre-subjective and post-subjective ratings to observe the choice-induced change. Results of the following simulation confirmed that no change of decision consistency was found in cases where no preference change exists (Fig. S1).

In the simulation, 24 hypothetical participants' data were generated: 28 word stimuli and 112 word pairs (trials) were used for our experiment. Following the method reported by Izuma and Murayama [2], we first assigned a true preference to each word stimulus of each participant. The true preference was assigned randomly by sampling from a normal distribution with mean 5.5 and standard deviation (*SD*) 2, as Izuma and Murayama reported[2]. For each participant and task, 112 pairs were generated randomly with the restriction that each term was used eight times. Then, a response series to 112 trials of decision task was generated: we added random noise (decision noise) to the true preference of each word stimulus. Then words with higher (decision-noise contaminated) preference from word pairs were selected. Decision noise was generated randomly from a normal distribution with mean 0 and *SD* 0, 0.25, 0.5, 0.75, 1, 1.25, 1.5, 1.75, 2, 2.25, or 2.5. We calculated the decision consistency score (for details see *Methods*), and the changes of decision consistency by subtracting that score of the first-half trials from the last-half trials. We conducted 10,000 simulation iterations for each magnitude of noise.

Figures S1(a) and S1(b) present mean results of 10,000 simulations. No change of decision consistency was found for any decision noise setting. These results indicate that decision consistency

is not changed when no preference change exists. Consequently, the change of decision consistency is unaffected by the difficulty pointed out by Chen and Risen[1].

Simulation 2

Moreover, we conducted an additional simulation (Simulation 2) to confirm that the increase of decision consistency is observed when choice-based learning (CBL) occurs. We used a simple CBL model in which the value of the chosen item ($Q_i^{chosen}(t)$) was increased (equation 1), whereas the value of the rejected item($Q_i^{rejected}(t)$) was decreased (equation 2) as follows (i is the index of the item; *t* is the index of the trial).

$$\begin{cases} Q_i^{chosen}(t+1) = Q_i^{chosen}(t) + \alpha \cdot (1 - Q_i^{chosen}(t)) & \text{(equation 1)} \\ Q_i^{rejected}(t+1) = Q_i^{rejected}(t) - \alpha \cdot (1 - Q_i^{rejected}(t)) & \text{(equation 2)} \end{cases}$$

Therein, α is the learning rate that determines how much the model updates the item value. α was varied from 0 to 0.7 at intervals of 0.05. The upper limit (0.7) was determined based on the 95% confidence interval (0.18–0.68) of the learning rate parameter estimate of the previous studies of CBL[3], which used computational model fitting of behavioral data. The range of the item value $Q_i(t)$ is restricted to values between 0 and 1. α is multiplied by $(1 - Q_i(t))$ to restrict $Q_i(t+1)$ between 0 and 1, after updating the item value. We generated hypothetical data almost identically to the method described for Simulation 1, except when the item value was between 0 and 1 (the range was approximately 1/10 of Simulation 1). The true preference was assigned randomly by sampling from a normal distribution with mean 0.55 and *SD* 0.1. Decision noise was generated randomly from a normal distribution with mean 0 and *SD* 0, 0.025, 0.05, 0.075, 0.1, 0.125, 0.15, 0.175, 0.2, 0.225, or 0.25.

Figure S1(c) presents mean results of 10,000 simulations by applying both equations 1 and 2. An increase of decision consistencies was observed when the learning rate (α) was neither 0 nor 0.7. When the learning rate was 0.7 and the decision noise was greater than 0.15, the change of decision consistency was around 0. These results indicate that the decision consistency is increased when CBL occurs without too high a learning rate and decision noise.

Figures S1(d) and S1(e) respectively present mean results of 10,000 simulations by applying only equations 1 or 2. In both results, the increase of decision consistency was observed except for the case in which the learning rate was 0. These results confirmed that the decision consistency increased when CBL occurs.

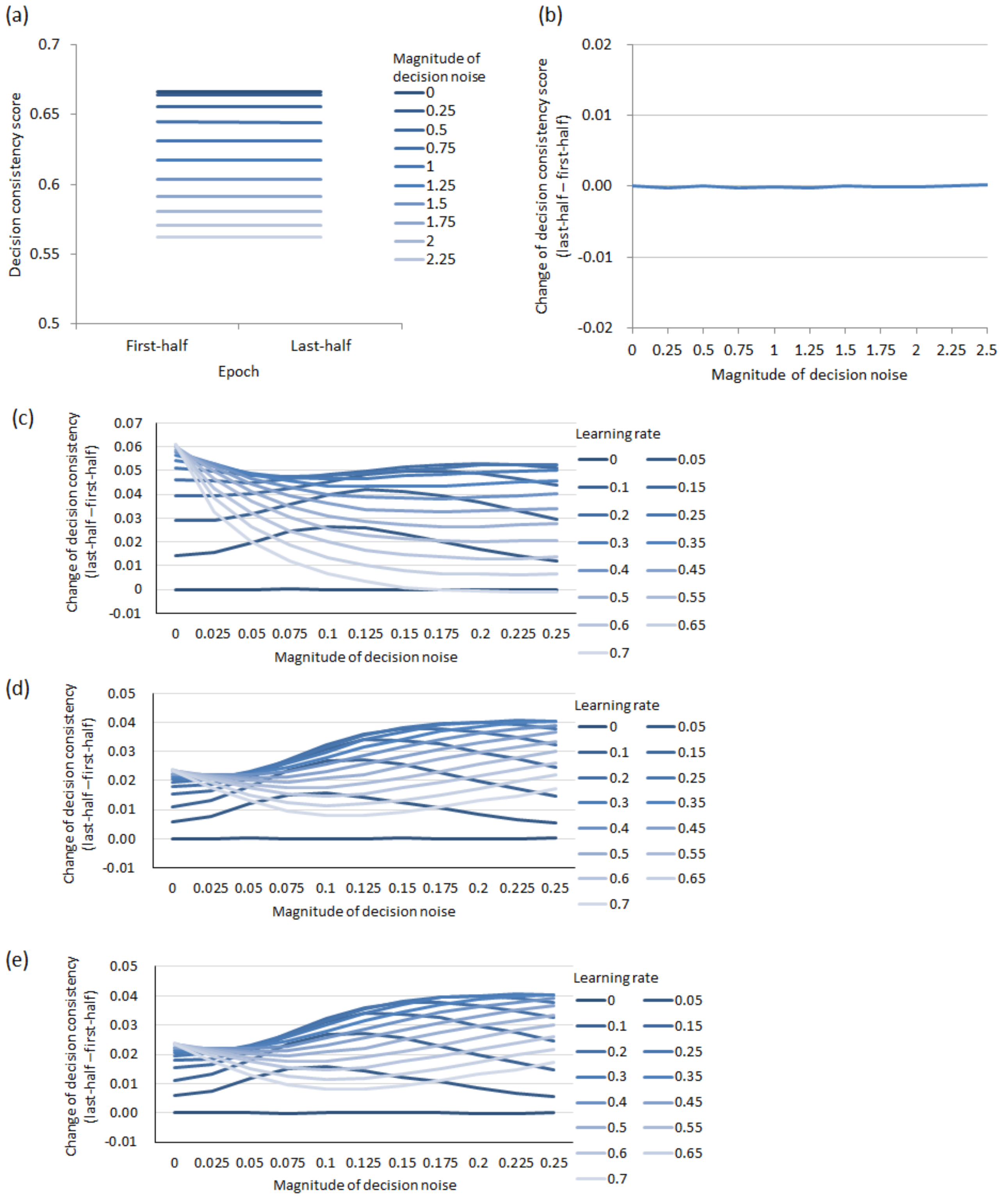

Figure S1. Simulation 1 results (replication = 10,000) of decision consistency for each epoch (a) and the change of decision consistency (b). (c) Simulation 2 results (replication = 10,000) of the change of decision consistency by application of equations 1 and 2. (d) Simulation 2 results (replication = 10,000) of the change of decision consistency by application of only the equation 1. (e) Simulation 2 results (replication = 10,000) of the change of decision consistency by the application of equation 2.

Supplemental Methods

Controls of stimuli

To confirm that the averaged annual salary does not covariate with the used-frequency of each occupation-related term, Google (http://www.google.jp) web page hits (collected on 10 July 2013) were used to estimate the used-frequency of each term, as in previous studies.[4–6] No correlation was found between the average annual salary and used-frequency (Pearson's r=0.14). A lack of correlation was confirmed between the average annual salary and the word length (Pearson's r=-0.12).

Trial-based decision consistency

To observe the change of decision consistency at an individual trial level, we calculated the index of the CBL at the trial level (trial-based decision consistency; Figure S2). The trial-based decision consistency score represents the rate of consistently chosen or rejected stimuli for each pair of consecutive trials including the same stimuli. To calculate the index, the consistently chosen or rejected stimuli were counted for each pair of consecutive trials including the same stimuli (e.g., first time trial including "Lawyer" and second time trial including "Lawyer"). For each pair of consecutive trials (e.g., first time - second time, second time - third time…), the stimuli that were consistently chosen or rejected were counted. Then that number was converted to a rate of consistently chosen or rejected stimuli (i.e., trial-based decision consistency) by dividing the total number of stimuli (i.e., 28).

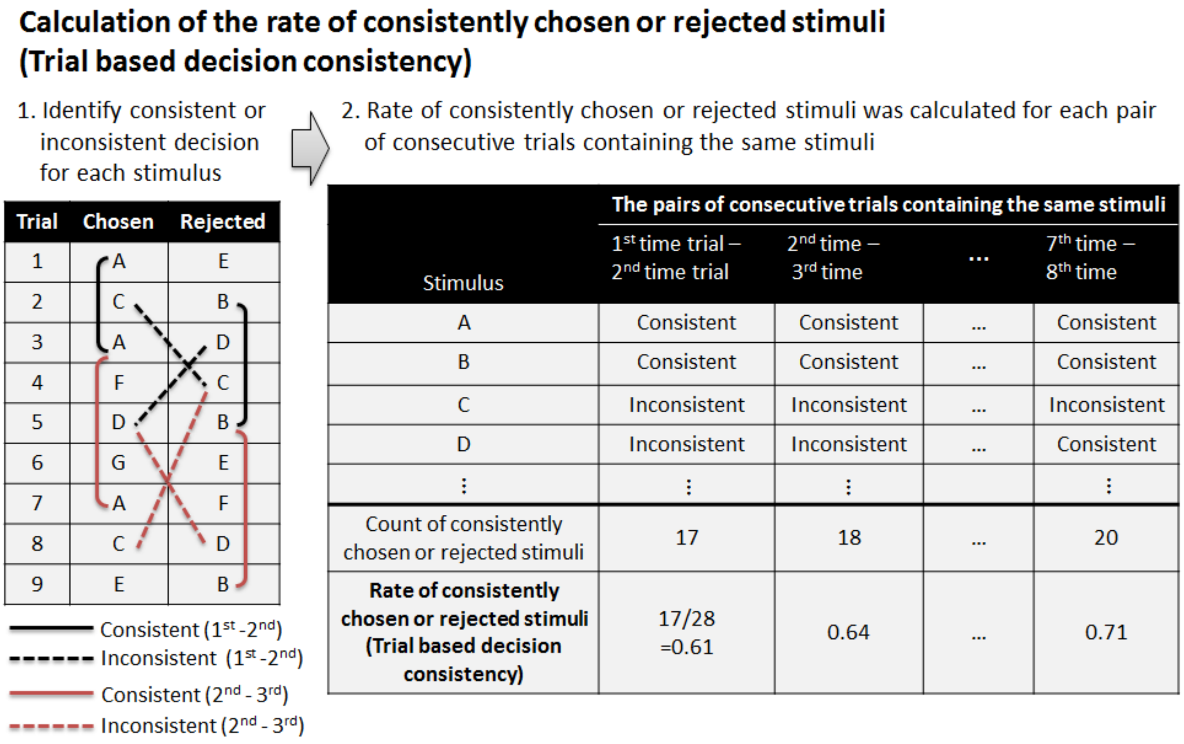

Figure S2. Schematic showing calculation of trial-based decision consistency.

Ratings for respective occupations and consistency between the pre-rating and decision making task (pre-rating – decision consistency)

Before judgment tasks, participants were asked to rate the two dimensions (preference and salary) using a computer-based visual analog scale for all occupation words. The following questions and scales were used for the ratings: Preference ("How much would you like to do the job?" 1 = not at all, 100 = very much) and salary ("How much pay is given for the following occupations?; 1 = very little, 100 = very much). The order used to rate these items was randomized across participants.

Pre-ratings for preference and salary were used to confirm whether participants' criteria for internally guided decision making (IDM) and externally guided decision making (EDM) differed. For this, we counted trials that are consistent between the rating value of each word stimulus and the judgment of the decision-making task. For example, for a case in which the participant rated occupation A as 100 (very much) and occupation B as 1 (not at all) regarding one's preference, and chose occupation A (100) compared to B (1) in the IDM (occupational preference judgment), we counted the trial as consistent. The sum of the number of consistent trials was divided by the total number of trials in each task. This index (pre-rating – decision consistency) represents how often participants' decisions were consistent with pre-ratings of the same dimension (i.e., preference or salary).

Average annual salary – decision consistency

Average annual salary – decision consistency represents how often participants' decisions were consistent with actual average annual salary based on a statistical survey by the Ministry of Health, Labour and Welfare of Japan. This index was calculated in the same way as the pre-rating – decision consistency. However, instead of the subjective pre-rating, we used data of a statistical survey of average annual salaries.

EEG recordings

EEG were recorded using 30 silver – silver chloride cup electrodes attached to an electrocap (Quik-Cap; NeuroScan), with electrodes placed at Fp1, Fp2, F7, F3, Fz, F4, F8, FT7, FC3, FCz, FC4, FT8, T7, C3, Cz, C4, T8, TP7, CP3, CPz, CP4, TP8, P7, P3, Pz, P4, P8, O1, Oz, and O2 according to extended International 10–20 Systems. Although the averages of C3 and C4 electrodes were used as the reference during on-line recording, all electrodes were later re-referenced to averaged earlobes. Blink and eye movements were monitored with electrodes above and below the left eye (vertical electrooculogram, VEOG) and at the right and left outer canthi of the eyes (horizontal electrooculogram, HEOG). The electrode impedance was maintained as less than 5 kΩ. The EEG and EOG signals were amplified with a bandpass of 0.0159–120 Hz, and were digitized at a 1,000 Hz sampling rate using an EEG recorder (EEG-1100; Nihon Kohden Corp., Tokyo, Japan).

Artifact rejection from EEG data

Epochs with irregular noise were identified and rejected using a computer algorithm and inferences from visual inspection.[7] Typical physiological artifacts such as eye blinks, eye movement, and muscle potentials were retained for the following independent component analysis (ICA).

Extended infomax ICAs were conducted to obtain 32 ICs from response-locked epochs in each participant. An equivalent current dipole was estimated for each IC (DIPFIT 2.2, EEGLAB plug-in using Fieldtrip toolbox). ICs representing typical physiological artifacts and electrode artifacts were identified by visual inspection of their time course data, multi-trial event-related potential (ERP) image plots, the power spectrum, scalp topography, and dipole. On average, 9.38 ICs were rejected from each participant's data. The remaining ICs were back-projected onto the scalp electrodes to obtain artifact-free EEG data.

Statistical analysis of group averaged ERSP

Regarding group averaged ERSP, we conducted the following comparisons of three types. First, we performed sample *t*-tests for ERSP in first and last-half trials of each task to examine whether the increased beta–gamma power after response was observed in each task and each epoch (i.e., the first-half or the last-half trials). Second, we conducted paired *t*-tests to compare ERSPs in first and last-half trials of each task to examine whether the beta–gamma power was altered with trial progress. Third, we conducted paired *t*-tests to compare ERSPs in IDM (preference judgment) and EDM (salary judgment) tasks of each epoch.

We performed a cluster-based permutation test[8] for each *t*-test to avoid multiple comparisons in the large time-frequency space. In step 1, we calculated the *t*-value for each pixel datum of the time-frequency window (-200 ms – 600 ms, 2–60 Hz) at FCz. Those were thresholded using uncorrected parametric *p*-values (p<0.05). Step 2, the bwconncomp Matlab function was applied to identify clusters in the thresholded map. The sum of the *t*-value in each cluster was calculated. Step 3, to generate the probability distribution of the sum of *t*-value under the null hypothesis, 2,000 iterations of the following three steps were conducted: First, the condition label (e.g., first vs. last trials) was shuffled randomly. Second, as with the steps 1 and 2 described above, we calculated the *t*-value for each pixel data. Those were thresholded using uncorrected parametric *p*-value (p<0.05). Third, we collected the largest sum of the absolute *t*-value in the cluster. The distribution generated by the iterations was used to calculate the critical value.

Statistical analysis of correlation analyses

As with the case with group-averaged ERSP comparison (*t*-tests), we performed a cluster-based permutation test [8] to avoid issues of multiple comparisons in the large time-frequency space. The procedure was almost identical to that of the case of *t*-tests: the differences were that we calculated Pearson's correlation coefficients (*r*-values) instead of *t*-values. Moreover, the mapping of

a variable to participants was shuffled randomly (instead of the condition label) for the 2,000 iterations. For this permutation test, the frequency range was limited to the beta–gamma band (14–60 Hz).

Phase-Amplitude cross-frequency coupling

For further exploratory analysis for the physiological feature of beta–gamma power after response in the first-half trials of the IDM (preference) task, we computed phase–amplitude couplings between the beta–gamma (25–40 Hz) power and theta–alpha phase (4–13 Hz) around 425 ms. The time window size was defined by the three cycles of lower frequency for phase. The measure of phase–amplitude coupling (PAC; Canolty et al., 2006) was calculated for each combination between the theta–alpha (for phase) and the beta–gamma (for power/amplitude) frequencies. The PAC is transformed to a normalized *z*-value (PACz) using permutation method [8]: we computed the PAC values 2,000 times by shuffling the power time series within trials. Then we used the mean and the standard deviation of that distribution of the PACs to calculate the *z*-value. For group level statistical analysis, permutation one-sample *t*-tests were used in the same manner as in the case of group-averaged ERSP analysis. For reference, the couplings in the other conditions (the last-half trials in the IDM task, and the first and the last-half trials of the EDM task) were calculated as they were in the first-half trials in the IDM task.

Supplemental Results

Trial-based decision consistency score

Results of this index are presented in Figure S3(a). Two-way repeated measures of ANOVA (2 tasks × 7 pairs of consecutive trials including the same stimuli) revealed marginally significant interaction ($F(6, 138)=2.14, p=0.053, \eta_g^2=0.04$). Post hoc analysis revealed a simple main effect of pairs of consecutive trials ($F(6, 138)= 2.88, p<0.05, \eta_g^2=0.09$) for the preference judgment task. Shaffer's test revealed that this index was increased significantly from third time – fourth time presented trials to fourth time – fifth time presented trials in the IDM (preference judgment). No significant increase of this index was found in the EDM (salary judgment). The index at the fourth time – fifth time presented trials and fifth time – sixth time presented trials in the IDM (preference judgment) were significantly larger than those in EDM (salary task) (Fs(6, 138)>5.72, ps<0.05, η_g^2s>0.09).

Different from the results of epoch-based decision consistency (Last-half – first half; Fig. 1(d)), no main effect of trials was found ($F(6, 138)=0.58, p=0.16, \eta_g^2=0.03$). A possible reason for the discrepancy is that the trial-based decision consistency can be affected strongly by the combination of stimulus: if participants selected "Lawyer" in the trial of "Lawyer" vs. "Designer" for preference judgment, the choice would be affected if the more preferred or less preferred

occupation was paired with "Lawyer", whether the "Lawyer" is chosen or rejected at the next opportunity. The combination of the two option was determined randomly across trials. Therefore, if no CBL exists, no change of epoch-based decision consistency would be observed (presented in Fig. 1s(a) and (b)). However, that effect from the combination of the two options functions as noise to decrease the statistical power to observe decision consistency (both in the epoch-based and trial-based decision consistency), and trial-based decision consistency would be affected strongly by that noise because fewer trials were used to calculate one index value.

Pre-rating decision consistency

Pre-rating decision consistency represents how often participants' decisions were consistent with pre-ratings of the same dimension (i.e., occupational preference or salary). The results of this index (see Fig. S3(b)) confirmed that criteria for the IDM and the EDM differed. Two-way repeated-measures ANOVA (two decision tasks (IDM, EDM) × two dimensions of pre-rating) revealed a significant main effect of task ($F(1,23)=8.56$, $p<0.01$, $\eta_g^2=0.028$), a significant main effect of dimension ($F(1,23)=10.57$; $p<0.01$, $\eta_g^2=0.099$), and significant interaction ($F(1,23)=169.85$; $p<0.001$, $\eta_g^2=0.69$). Post-hoc tests of the interaction revealed that the index was higher in the IDM (preference judgment) task than in the EDM (salary judgment) tasks ($p<0.001$) in the dimensions of preference. In the dimension of salary, the index was higher in the EDM (salary judgment) task than in the IDM (preference judgment) task ($p<0.001$). These results indicate that participants differentiated the decision criteria for the two decision-making task types.

Reaction time

Figure S3(c) presents a summary of mean reaction times (RTs) and shows that RTs were shorter in the last-half trials than in first-half trials both in IDM (preference) and EDM (salary) tasks. Consistent with this observation, two-way repeated-measures ANOVA (two tasks (IDM, EDM) × two epochs (first and last-half trials)) revealed a significant main effect of epoch ($F(1,23)=10.80$, $p=0.003$, $\eta_G^2=0.02$). No significant main effect of task ($F(1,23)=1.88$, $p=0.18$, $\eta_G^2=0.004$) and interaction ($F(1,23)=0.02$, $p=0.89$, $\eta_G^2=0.00$) was found.

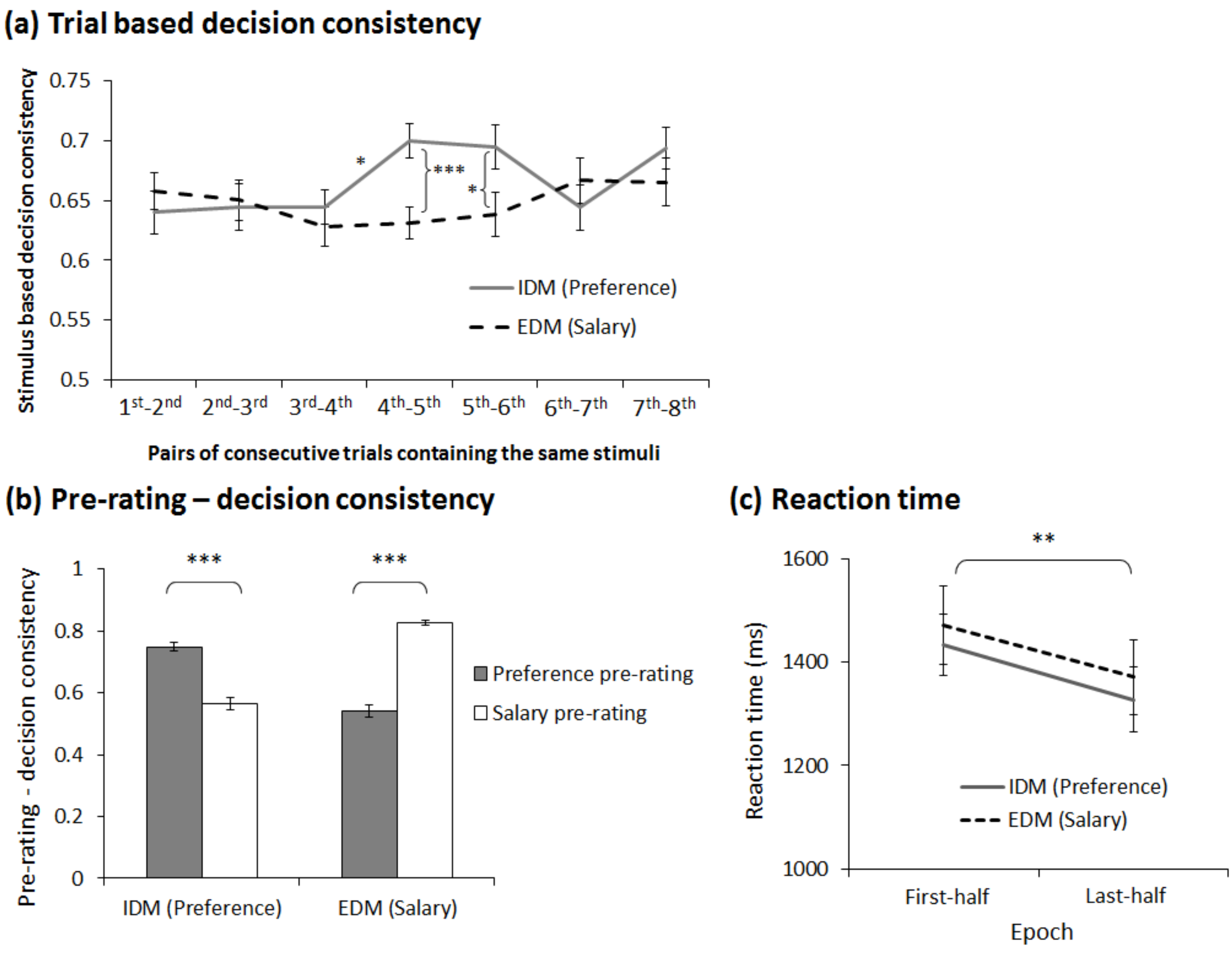

Figure S3. Supplemental behavioral data. (a) Trial-based decision consistency for each decision making task. IDM denotes internally guided decision-making. EDM denotes externally guided decision-making. * denotes significant difference (p<0.05). *** denotes significant difference (p<0.001). (b) Pre-rating decision consistency for each combination of pre-rating and decision-making task. *** denotes significant difference (p<0.001). (c) Reaction times for the first-half and the last-half trials in the IDM (preference) and EDM (salary) tasks. ** denotes a significant main effect of epoch (p<0.005). Error bars represent standard errors.

Correlation between the post-response beta-gamma power during the first-half trials and change of decision consistency using pre-stimulus baseline corrected ERSP data

In the main text, we avoided using the pre-stimulus baseline because post-response activities (i.e., the target activities of the present study) of the preceding trial can contaminate the pre-stimulus baseline. However, if the observed correlation during the post-response duration (Fig. 2a) is not caused by the pre-response activities, then similar patterns of post-response correlation are expected to remain after applying pre-stimulus baseline (-442 – 0 ms), even if the effect size is decreased. The pre-stimulus baseline ERSP was calculated using the same setings with response-locked ERSP. Although no significant cluster was found using permutation tests, positive correlations after response were observable in IDM after applying the pre-stimulus baseline (see Fig. S4). In addition, when extracting the averaged power from the time-frequency window of interest (350–500

ms and 25–40 Hz), significant correlation with the change of decision consistency was observed (r=0.46, p<0.05). Although the effect size was decreased, which might result from the contamination of post-response activities of the preceding trial in the pre-stimulus baseline, these results confirm that the post-response beta-gamma activities in the first-half trials correlate with the change of decision consistency in IDM.

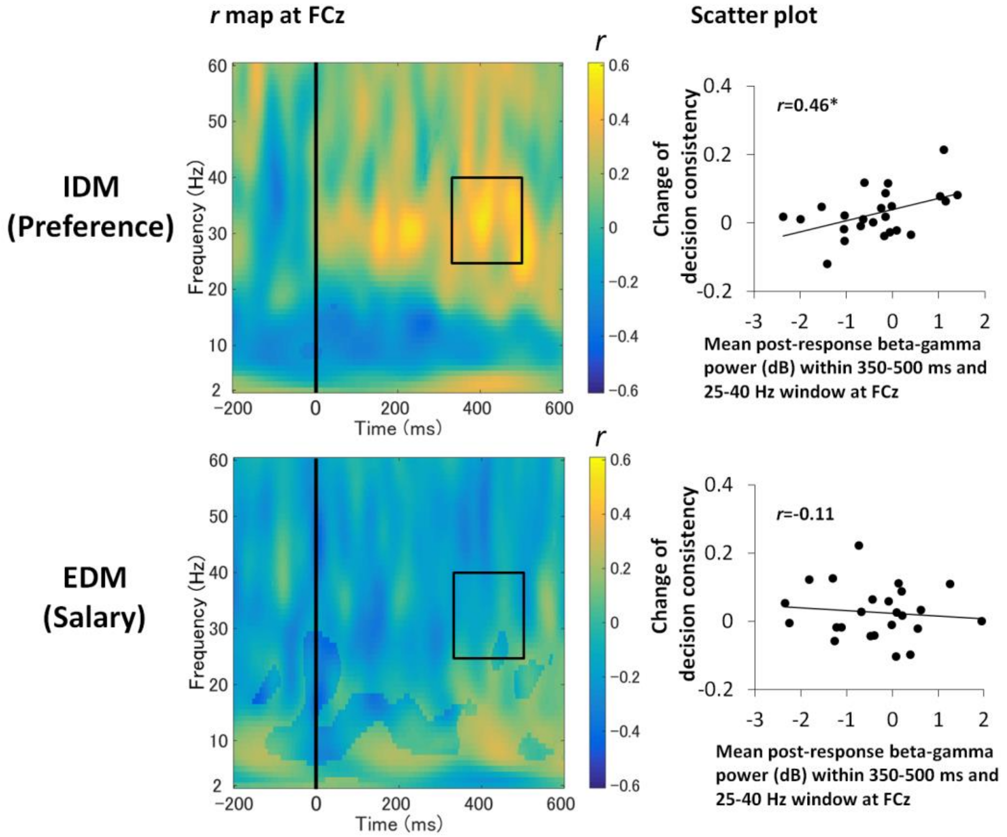

Figure S4. Correlation results between the response-locked event-related spectral perturbations (ERSP) at FCz for the first-half trials and the change of decision consistency (last-half trials – first-half trials) for each decision-making task. Pre-stimulus baseline (-442 – 0 ms) activities were used for the baseline to calculate ERSP. Scatter plots between the change of decision consistency and mean beta–gamma power within the time-frequency window of interest (350–500 ms and 25–40 Hz) are shown on the right side. * denotes a significant main effect of epoch (p<0.05).

Group averaged ERSP

Figure S5 presents response-locked ERSP for each task (IDM and EDM tasks) and each epoch (first-half and last-half trials) at FCz, significant clusters from one sample permutation t-tests, and scalp topographies of mean ERSP within the significant cluster. In every four conditions, the permutation one sample t-tests yield significant beta–gamma power increase after around 400 ms (cluster t-value sum= 4098.69, cluster count = 1057, corrected p<0.05 for first-half trials of IDM task; cluster t-value sum= 5565.87, cluster count = 1522, corrected p<0.05 for last-half trials of IDM

task; cluster *t*-value sum= 4024.64, cluster count = 1274, corrected $p<0.05$ for first-half trials of EDM task; cluster *t*-value sum= 5689.70, cluster count = 1465, corrected $p<0.05$ for last-half trials of EDM task). Furthermore, a significant theta power decrease after around 200 ms (cluster *t*-value sum= 5509.61, cluster count = 1028, corrected $p<0.05$ for first-half trials of IDM task; cluster *t*-value sum= 8093.47, cluster count = 1760, corrected $p<0.05$ for last-half trials of IDM task; cluster *t*-value sum= 7359.06, cluster count = 1586, corrected $p<0.05$ for first-half trials of EDM task; cluster *t*-value sum= 3370.11, cluster count = 807, corrected $p<0.05$ for last-half trials of EDM task) was found.

Although we conducted permutation paired *t*-tests for first-half vs. last-half trials in each task and IDM vs. EDM in each epoch, no significant cluster was found.

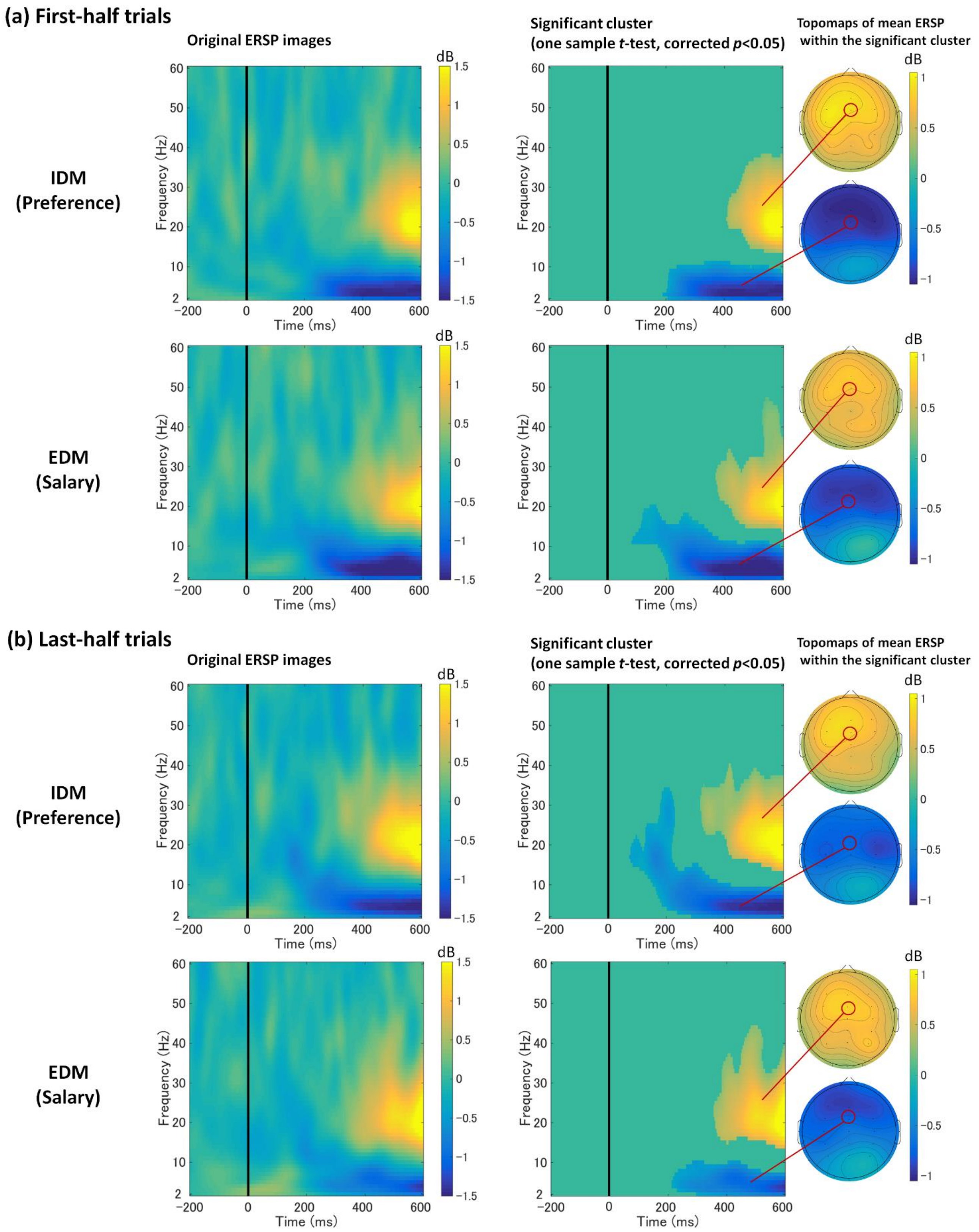

Figure S5. Response-locked event-related spectral perturbations (ERSP) images at FCz for the first-half and the last-half trials in the IDM (preference) and EDM (salary) judgment tasks. Scalp topographies of mean power within each significant cluster are shown on the right side. IDM denotes internally guided decision-making. EDM denotes externally guided decision-making.

Phase–Amplitude coupling

Figure S6 presents phase–amplitude coupling between the theta–alpha phase and beta–gamma power around 425 ms after response for each task (IDM and EDM tasks) and each epoch (first-half and last-half trials) at FCz. No significant coupling was found after applying corrected $p<0.05$. Although conducted correlation analyses were also between the PACz in the first-half trial and the change of decision consistency in the IDM and EDM tasks, no significant correlation was found after applying corrected $p<0.05$.

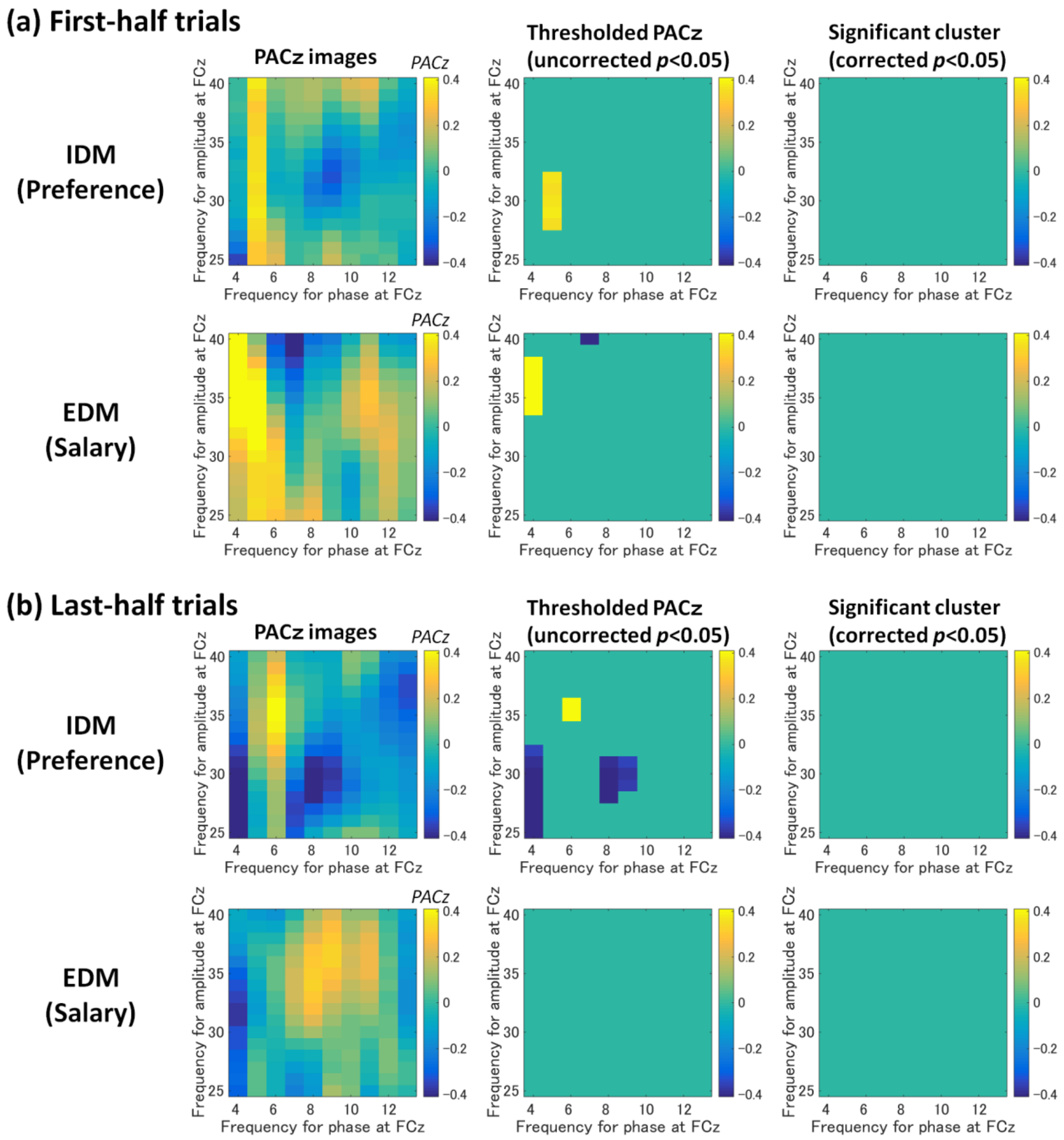

Figure S6. Results of phase–amplitude coupling around 425 ms after response at FCz for each condition. IDM denotes internally guided decision-making. EDM denotes externally guided decision-making.

Supplemental References

1. Chen, M. K. & Risen, J. L. How choice affects and reflects preferences: revisiting the free-choice paradigm. *J. Pers. Soc. Psychol.* **99,** 573–594 (2010).

2. Izuma, K. & Murayama, K. Choice-induced preference change in the free-choice paradigm: a critical methodological review. *Front. Psychol.* **4,** 41 (2013).

3. Akaishi, R., Umeda, K., Nagase, A. & Sakai, K. Autonomous Mechanism of Internal Choice Estimate Underlies Decision Inertia. *Neuron* **81,** 195–206 (2014).

4. Caldwell-Harris, C. L. & Morris, A. L. Fast pairs: a visual word recognition paradigm for measuring entrenchment, top-down effects, and subjective phenomenology. *Conscious. Cogn.* **17,** 1063–1081 (2008).

5. Myers, J., Huang, Y. & Wang, W. Frequency effects in the processing of Chinese inflection. *J. Mem. Lang.* **54,** 300–323 (2006).

6. Nakao, T., Bai, Y., Nashiwa, H. & Northoff, G. Resting-state EEG power predicts conflict-related brain activity in internally guided but not in externally guided decision-making. *Neuroimage* **66,** 9–21 (2013).

7. Delorme, A., Sejnowski, T. & Makeig, S. Enhanced detection of artifacts in EEG data using higher-order statistics and independent component analysis. *Neuroimage* **34,** 1443–1449 (2007).

8. Cohen, M. X. *Analyzing Neural Time Series Data*. (MIT Press, 2014).

9. Canolty, R. T. *et al.* High Gamma Power Is Phase-Locked to Theta Oscillations in Human Neocortex. *Science* **313,** 1626–1628 (2006).
